# Non-affinity in multi-material mechanical metamaterials

**DOI:** 10.1038/s41598-020-67984-6

**Published:** 2020-07-13

**Authors:** M. J. Mirzaali, H. Pahlavani, E. Yarali, A. A. Zadpoor

**Affiliations:** 1 Department of Biomechanical Engineering, Faculty of Mechanical, Maritime, and Materials Engineering, Delft University of Technology (TU Delft), Mekelweg 2, 2628 CD Delft, The Netherlands; 2 School of Mechanical Engineering, College of Engineering, University of Tehran, Tehran, Iran

**Keywords:** Engineering, Mechanical engineering, Structural materials, Composites, Materials science, Soft materials, Polymers

## Abstract

Non-affine deformations enable mechanical metamaterials to achieve their unusual properties while imposing implications for their structural integrity. The presence of multiple phases with different mechanical properties results in additional non-affinity of the deformations, a phenomenon that has never been studied before in the area of extremal mechanical metamaterials. Here, we studied the degree of non-affinity, $\Gamma$, resulting from the random substitution of a fraction of the struts,${\rho }_{h}$, that make up a lattice structure and are printed using a soft material (elastic modulus = ${E}_{s}$) by those printed using a hard material (${E}_{h}$). Depending on the unit cell angle (i.e., $\theta$ = 60°, 90°, or 120°), the lattice structures exhibited negative, near-zero, or positive values of the Poisson’s ratio, respectively. We found that the auxetic structures exhibit the highest levels of non-affinity, followed by the structures with positive and near-zero values of the Poisson’s ratio. We also observed an increase in $\Gamma$ with $\frac{{E}_{h}}{{E}_{s}}$ and ${\rho }_{h}$ until $\frac{{E}_{h}}{{E}_{s}}$ =10^4^ and ${\rho }_{h}$= 75%-90% after which $\Gamma$ saturated. The dependency of $\Gamma$ upon ${\rho }_{h}$ was therefore found to be highly asymmetric. The positive and negative values of the Poisson’s ratio were strongly correlated with $\Gamma$. Interestingly, achieving extremely high or extremely low values of the Poisson’s ratio required highly affine deformations. In conclusion, our results clearly show the importance of considering non-affinity when trying to achieve a specific set of mechanical properties and underscore the structural integrity implications in multi-material mechanical metamaterials.

## Introduction

A simple mechanical load (e.g., uniaxial compression, tension, or shear) applied to a geometrically simple (e.g., square-shaped) piece of what is traditionally considered a material (e.g., metals, polymers) leads to a simple deformation that is highly predictable at large enough length scales and is homogeneously distributed within the material. Such a homogenous deformation is formally called an ‘affine’ deformation and can be fully described using an affine transformation (i.e., a linear transformation plus a rigid body translation) applied to the coordinates of the points constituting the material^1,2^.

All this simplicity, predictability, and homogeneity may be lost when a simple mechanical load is applied to an architected material. Architected materials^3^, which are sometimes referred to as mechanical metamaterials^4–6^, possess complex small-scale geometries that are engineered to give rise to unusual mechanical properties at the macroscale. In a way, the whole point of rationally designing^7^ the small-scale geometry of architected materials, may be to break the affinity of the deformations in an exact way so as to achieve unusual macroscale properties. Non-affine deformations can, for example, be exploited to achieve negative values of the Poisson’s ratio^8,9^, action-at-a-distance actuation behaviors^10^, and independent tailoring of the elastic properties^11^. Some other functionalities of mechanical metamaterials such as shape morphing^12,13^ are ‘per definition’ dependent on the non-affinity of the induced deformation. Non-affine deformations can also be observed in other systems. For example, random networks^14–17^ that are found in biological systems such as the filamentous proteins that make up the cytoskeleton and extracellular matrix^18–22^, as well as flexible polymer networks^23^ and polymer hydrogels^24^ exhibit highly non-affine deformations.

From the structural integrity viewpoint, however, non-affine deformations could be troublesome, as they may give rise to stress concentrations and, thus, decreased fatigue lives and premature failures. It is, therefore, crucial to understand the non-affinity of deformations in architected materials because both advanced functionalities and structural performance of these materials are dependent on the proper management of non-affine deformations. Even though non-affine deformations can be studied at different length scales^15,25–27^, the most relevant scale in the case of mechanical metamaterials is the length scale of the constituting structural elements (i.e., struts)^28^. We will, therefore, be focusing on this length scale.

Non-affine deformation can be characterized in terms of a degree of non-affinity ($\Gamma$) or non-affine correlation functions^14,29,30^. The degree of non-affinity is a scalar parameter that depends on the length scale^31^ and applied strain^15,19,21^. There are also other metrics of non-affinity that are based on strain energy^15,19,31,32^ or a comparison of the local deformations with affine deformations^14,29,30,33–39^.

The degree of non-affinity is an important determinant of the inhomogeneous deformation of metamaterials. However, its relationship with the elastic properties (e.g.*,* elastic modulus and Poisson’s ratio) of mechanical metamaterials remains elusive. Recent advances in multi-material additive manufacturing (also called 3D printing) techniques have enabled the fabrication of ‘multi-material’ mechanical metamaterials^40–42^ whose unusual properties and advanced functionalities are as much dependent on the spatial distribution of multiple phases with different mechanical properties as they are on the small-scale geometrical design of the constituting unit cells. Essentially, the complex distributions of the multiple phases are alternative ways of creating non-affine deformations so as to expand the range of achievable macroscale properties^41^. From the structural integrity viewpoint, the presence of multiple phases with highly different mechanical properties creates stress concentrations that underscore the importance of studying the non-affinity of the deformations even more. However, non-affine deformations in multi-material mechanical metamaterials have never been studied before.

Here, using computational models and experimental tests, we studied the non-affinity of the deformations taking place in a special class of multi-material mechanical metamaterials that are made from two distinct phases, namely a hard phase and a soft phase. We aimed to separate the non-affinity caused by the presence of multiple materials from that of geometrical design.

For this purpose, the degree of non-affinity was determined by comparing the deformations taking place in the multi-material mechanical metamaterials (i.e.*,* heterogeneous structures) with those of reference materials with monolithic properties (i.e.*,* homogeneous structures) using computational models. The properties of the homogeneous structure equivalent to each heterogeneous structure were calculated using the rule-of-mixture, the properties of the soft and hard phases, and their ratios. Moreover, three different structures with cell angles of ${60}^{o}$, ${90}^{o}$, and ${120}^{o}$ and with different mechanical properties were fabricated using an advanced multi-material 3D printing technique. Finally, the elastic properties of multi-material mechanical metamaterials and the degree of non-affinity were quantified and discussed in both quantitive and qualitative terms.

## Materials and method

**Figure 1:**
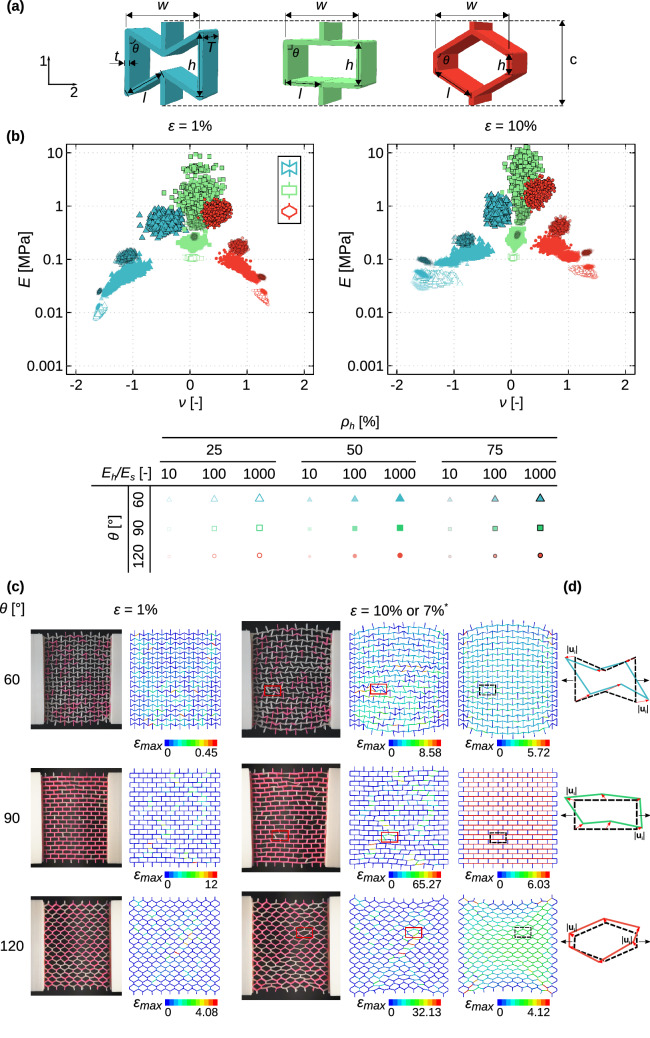
The three types of unit cells used in this study that represent negative (re-entrant, $\theta =60^\circ$), near-zero (orthogonal, $\theta =90^\circ$), and positive (honeycomb, $\theta =120^\circ$) values of the Poisson’s ratio (**a**). The other geometrical parameters of the lattice structures are presented in Table S1 of the supplementary document. The duos of the elastic modulus and Poisson’s ratio calculated from the numerical simulations at two levels of the applied strain (i.e.*,* 1% and 10%) (**b**). The multi-material 3D printed specimens were mechanically tested under tensile loading and were compared with the finite element simulations (**c**). The quantitative data pertaining to this comparison are presented in Table S2 of the supplementary document. For the specimen with $\theta =60^\circ$, the applied strain was 10%, while it was 7% for the specimens with $\theta =90^\circ , 120^\circ$. The insets in subfigure (**c**) show the maximum strain distribution. Subfigure (**c**) shows the deformation of a homogenous lattice with an elastic modulus equal to the rule-of-mixture combination of the elastic moduli of the hard and soft phases. $|{u}_{i}|$ in (**d**) stands for the difference between the deformation of the *i*th node of a homogeneous lattice structure and the deformation of the exact same node in a multi-material specimen.

We used already existing geometrical designs^8,43^ to create our mechanical metamaterials. The specimens were fabricated based on three types of unit cells with negative ($$\theta =60^\circ $$), near-zero ($$\theta =90^\circ $$), and positive ($$\theta =120^\circ $$) values of the Poisson’s ratio (Fig. 1a). The dimensions of the unit cells ($$w,c)$$ and the overall dimensions of the structures ($$W,C)$$ were kept constant in all designs. The angle ($$\theta $$) changed the height ($$h$$) and length ($$l$$) of the unit cells. Similar in-plane ($$t$$) and out-of-plane ($$T$$) thicknesses were considered for the unit cells with different angles. The design parameters are listed in Table S1 (supplementary document).

The geometry of each lattice structures was created in Matlab (R2017b) and was then used as an input file for numerical simulations in a nonlinear finite element solver (Abaqus Standard 6. 14). We used the linear Timoshenko beam elements (B21) with a rectangular cross-section because these elements can capture axial deformations, bending, and shear. We assumed a plane stress condition in our computational models. The constitutive behavior of the soft phase was described using a hyperelastic material model (Neo-Hookean, ${C}_{10}=0.106$ MPa, ${D}_{1}=0.03$ MPa^−1^). We also used a hyperelastic material model for the hard phase and adjusted the parameters of the model correspondingly. For example, for the hard phase with 100 times stiffer elastic properties the Neo-Hookean material parameters were adjusted assuming the following parameters: ${100\times C}_{10}=10.6$ MPa, $\frac{{D}_{1}}{100}=0.0003$ MPa^−1^.

The multi-material lattice structures were created by considering multiple levels of the elastic modulus of the hard phase, ${E}_{h}$, to that of the soft phase, ${E}_{s}$ (i.e., $\frac{{E}_{h}}{{E}_{s}}$= 10^1^, 10^2^,10^3^). To spatially distribute the hard phase in the lattice structures, we chose three levels of the ratio of the volume of the hard phase (i.e., ${\rho }_{h}=$ 25%, 50%, and 75%). A random process was then used to assign the hard phase to randomly select struts of the unit cells constituting the lattice structures. First, a vector containing the random permutation of numbers from 1 to the total number of struts of the structure was generated. Then, a percentage of the first elements of the vector, equaling ${\rho }_{h}$, were selected. The selected elements specified to which struts the hard phase was to be assigned.

For the special case of $\frac{{E}_{h}}{{E}_{s}}=1000$, we extended our simulations by considering a wider range of ${\rho }_{h}$ values (i.e.*,* ${\rho }_{h}=$ 1%, 5%, 25%, 50%, 75%, 90%, 95%, and 99%). We also performed further numerical simulations for $\frac{{E}_{h}}{{E}_{s}}$ values of 10^4^, 10^5^, and 10^6^, while keeping ${\rho }_{h}$ constant at 50%. For every above-mentioned combination of the design parameters, we performed 1,000 simulations, resulting in a total of 51,000 simulations. This means that, for each batch of 1,000 simulations, the geometry, ${\rho }_{h}$ and $\frac{{E}_{h}}{{E}_{s}}$ were kept constant among the specimens while the distribution of the hard phase within the structure was modified.

A uniaxial displacement-controlled stretch test in the direction 2 (Fig. 1a) was simulated in all models. Two reference points were defined on the right and left sides of the lattice structure, which were kinematically coupled with the corresponding nodes of the structure (Figure S3 of the supplementary document). A displacement boundary condition (corresponding to 10% strain) was applied to one reference point, while constraining all the degrees of freedom of the other reference point (Figure S3 of the supplementary document).

The normal stress, $\sigma =\frac{F}{A}$, was defined as the ratio of the reaction force, $F$, to the initial cross-section area, $A=C\times T$. The longitudinal strain, ${\varepsilon }_{22}=\frac{\delta W}{W}$, was calculated as the ratio of the displacement along the direction 2, $\delta W$, to the initial length of the structure in that direction, $W$. The elastic modulus, $E$, was computed as the instantaneous slope of the stress–strain curve. The Poisson’s ratio was calculated as $\nu =-\frac{{\varepsilon }_{11}}{{\varepsilon }_{22}}$, where ${\varepsilon }_{11}$ is the lateral strain measured by the average of the displacements taking place in the direction 1 (${U}_{i1}$) with respect to the transverse length of the structure ($C=n \times c$) (i.e., ${\varepsilon }_{11}=\frac{\sum_{i=1}^{n}{U}_{i1}}{C}$, where $n$ is the total number of nodes at the lateral side of the structure along the direction 1).

The degree of non-affinity was defined as^29,31^ $\Gamma =\frac{1}{{{\varepsilon }_{22}}^{2}N}{\sum_{i=1}^{N}({u}_{i}^{non-affine}-{u}_{i}^{affine})}^{2}$ where $N$ is the total number of nodes in the structure, ${u}_{i}^{non-affine}$ is the local displacement of the *i*th node of the multi-material structure, and ${u}_{i}^{affine}$ is the corresponding displacement of the *i*th node of a corresponding single-material lattice structure (Fig. 1d). The elastic modulus of that single material was determined as the rule-of-mixture combination of the elastic moduli of the phases constituting the corresponding multi-material lattice structure (i.e., ${E}^{^{\prime}}=\frac{{\rho }_{h}\times {E}_{h}+{\rho }_{s}\times {E}_{s}}{{\rho }_{h}+{\rho }_{s}}$).

For our experimental study, we selected three representative cases for each angle of the unit cells. We manually segmented their geometry in Solidworks to create different hard and soft phases, which were later converted into STL (standard tessellation language) files. The STL files were then inputted into a multi-material 3D printer (Object500 Connex3, Stratasys), which works on the basis of material jetting (the Polyjet technology) and were directly 3D printed using commercially available materials, namely VeroCyan (hard phase, RGD841, shore hardness (D) = 83–86) and Agilus30 Black (soft phase, FLX985, shore hardness (A) = 30–35) (Fig. 1c). The Young’s modulus of the hard and soft phases were respectively 726.36 ± 59.77 MPa and 0.60 ± 0.05 MPa^44,45^. The selection of the materials was made to achieve $\frac{{E}_{h}}{{E}_{s}}\approx 1000$ (see Fig. 1 and Table S2 of the supplementary document for more information). To attach the specimens to the mechanical testing machine, a gripping system and pins were designed and additively manufactured using a fused deposition modeling (FDM) 3D printer (Ultimaker 2 + , Geldermalsen, The Netherlands) from polylactic acid (PLA) filaments (MakerPoint PLA 750 gr Natural). Monotonic uniaxial tensile tests were performed under displacement control (stroke rate = 2 mm/min) using a LLOYD instrument (LR5K, load cell = 100 N) mechanical testbench. The time, force, and displacement were recorded at a sampling rate of 20 Hz. The stress and strain were obtained correspondingly by dividing the force to the initial cross-section area and dividing the displacement to the initial free length of the specimens. The stiffness of the structure was measured from the stiffest slope of the stress–strain curve. Using a digital camera, the deformations of the specimens were captured, which were then used to calculate the Poisson’s ratio. We manually positioned a couple of points at the borders of the specimens in the digital images. We manually positioned 20 points around the periphery of the specimens captured in the digital images. We developed a Matlab code to trace the movement of individual points in those images. The Poisson’s ratio was calculated based on the changes in the coordinates of those points during the deformation.We repeated the mechanical tests for each specimen three times.

## Results and discussion

A wide range of the elastic moduli (i.e., 0.1–10 MPa) and Poisson’s ratios (i.e., − 1.6 to 1.4) could be obtained using the multi-material design approach followed in the current study (Fig. 1b). The duos of the elastic modulus and Poisson’s ratio (at 1% strain) corresponding to the auxetic (i.e., $\theta =60^\circ$) and honeycomb (i.e., $\theta =120^\circ$) structures approached the values resulting from the orthogonal unit cells (i.e., $\theta =90^\circ$) as ${\rho }_{h}$ increased (Fig. 1b). A similar trend was observed for the higher values of the applied strain (i.e., 10%) (Fig. 1b).

The deformation patterns and the elastic properties predicted using our numerical simulations agreed with the experimentally observed deformation patterns and experimentally determined values of the elastic modulus and Poisson’s ratio (Fig. 1c, and Table S2 in the supplementary document). The small differences between the numerical and experimental results could be due to the imperfections induced during the manufacturing process as well as the pre-stretching of the soft ligaments when attaching the specimens to the clamps. We also performed a mesh sensitivity analysis for the models shown in Fig. 1c. Each strut in our reference computational models consisted of one element. For the mesh sensitivity analysis, we doubled the number of elements per strut. Then, we compared the values of elastic modulus and Poisson's ratio obtained from the models with finer mesh and reference models. That comparison resulted in less than 3% difference. We also used higher-order 2D elements (B22) in our computational models. That resulted in less than 4% difference in the values of the elastic modulus and Poisson's ratios as compared to our reference model.

A number of unit cells showed very clear non-affine deformations as compared to geometrically identical lattice structures made from a single material (Fig. 1c, right side). Similar non-affine deformations were observed in our experiments and captured by our simulations (Fig. 1c-d).

**Figure 2:**
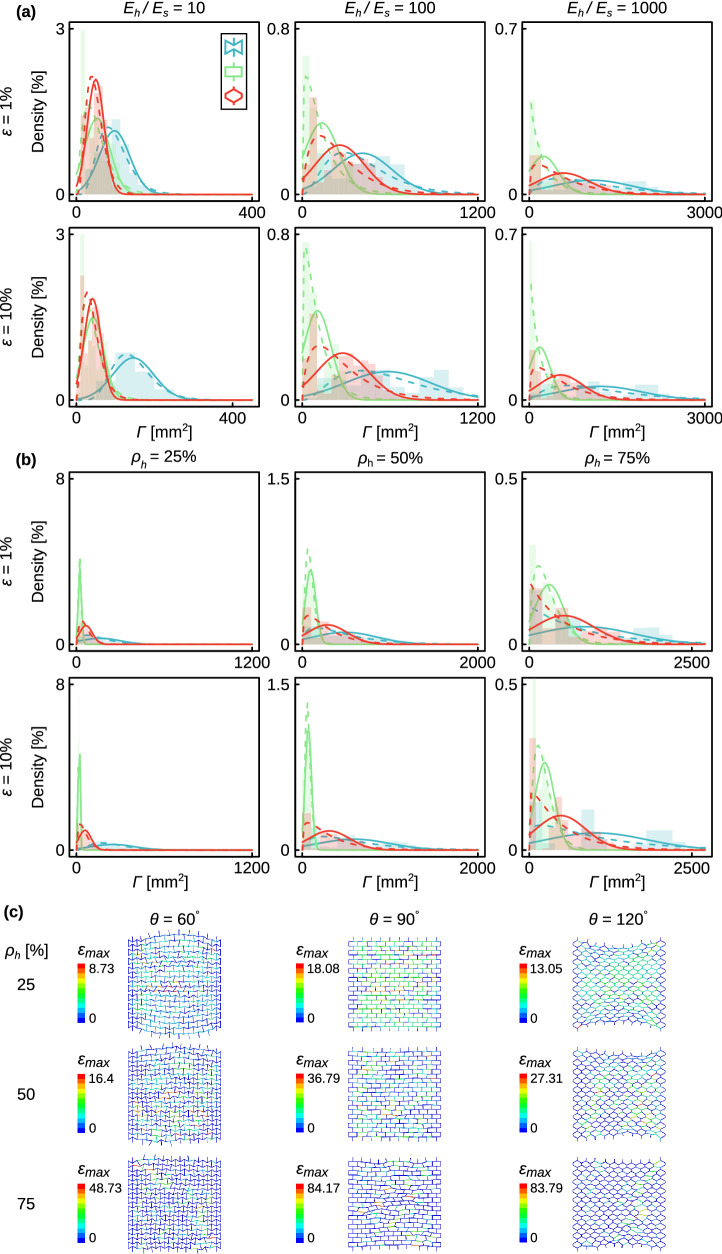
The Gaussian (solid lines) and gamma distributions (dashed lines) describing the change in the degree of non-affinity as functions of $\frac{{E}_{h}}{{E}_{s}}$ with pooled data (i.e.*,* ${\rho }_{h}=$ 25%, 50% and 75%) (**a**) and ${\rho }_{h}$ with pooled data ($\frac{{E}_{h}}{{E}_{s}}=$ 10, 100, and 1,000) (**b**). The parameters of these distributions are listed in Table S3 and S4 of the supplementary document. The degree of non-affinity for the representative cases shown in (**c**) is equal to the mean value of the corresponding group with $\frac{{E}_{h}}{{E}_{s}}=1000$ at 10% strain. The design parameters for each of the specimens presented in (**c**) are listed in Table S5 of the suppelementary document. The color bars in subfigure (**c**) show the maximum strain distribution in %.

For the same values of ${\rho }_{h}$ and $\frac{{E}_{h}}{{E}_{s}}$, the auxetic structures (i.e., $\theta =60^\circ$) always showed the maximum mean values of $\Gamma$, which were up to several times higher than those corresponding to the honeycomb (i.e., $\theta =120^\circ$) and orthogonal (i.e., $\theta =90^\circ$) lattice structures (Fig. 2a, Table S3 and Figure S1a of the supplementary document). Except for the case where the hard phase was not much stiffer than the soft phase (i.e., $\frac{{E}_{h}}{{E}_{s}}=10$), the honeycomb lattice structures exhibited a higher degree of non-affinity as compared to the orthogonal ones (Fig. 2a, Table S3 and Figure S1a of the supplementary document). There were significant overlaps between the range of the $\Gamma$ values found for the lattice structures with different values of ${\rho }_{h}$ (Fig. 2b, Table S4 and Figure S1b of the supplementary document). This observation suggests that the degree of non-affinity is more dependent on how the hard phase is distributed in the lattice than on the amount of the hard phase (Fig. 2b, Table S4 and Figure S1b of the supplementary document). Inspecting the deformations exhibited by the different types of the lattice structures clearly showed that those based on the re-entrant unit cell were more susceptible to the inhomogeneous deformations that result from the presence of high-stiffness struts (Fig. 2c). This is expected given the fact that the deformation of the re-entrant unit cell is dominated by the high stresses concentrated around its sharp corners, whereas stresses are generally more homogeneously distributed in the honeycomb and particularly orthogonal unit cells where the stress gradients within one single unit cell are relatively low (see Figure S4 of the supplementary document). Moreover, performing thousands of simulations with the random distribution of the hard phase within the lattice structure allows for determining the envelope within which the degree of non-affinity could change for a given value of ${\rho }_{h}$. Therefore, this envelope shows the possible range within which degree of non-affinity can change by the different spatial distribution of the hard phases in the lattices.

**Figure 3:**
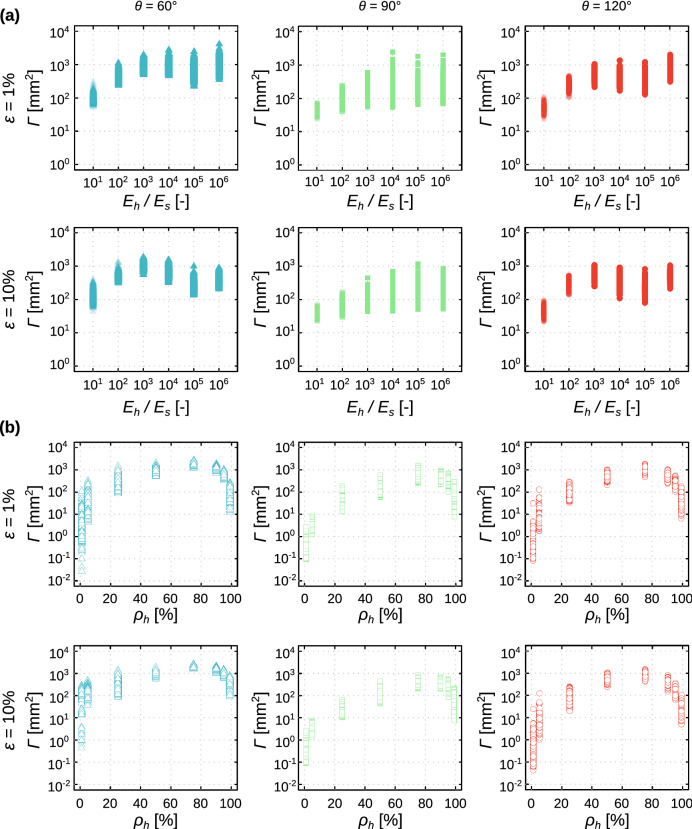
(**a**) The changes in the degree of non-affinity for different values of $\frac{{E}_{h}}{{E}_{s}}$ and for the three different types of the unit cell geometries (i.e., $\theta =60^\circ , 90^\circ , 120^\circ$) but the same value of ${\rho }_{h}=$ 50%. (**b**) The change in the degree of non-affinity for different hard volume fractions and for three types of unit cell geometries (i.e., $\theta =60^\circ , 90^\circ , 120^\circ$ with $\frac{{E}_{h}}{{E}_{s}}=1000$).

The degree of non-affinity initially increased with $\frac{{E}_{h}}{{E}_{s}}$ regardless of the type of the unit cell until $\frac{{E}_{h}}{{E}_{s}}$ =10^4^ after which it saturated (Fig. 3a-top). A hard phase with a higher stiffness value disrupts the stress flow to a greater extent than a hard phase with a lower level of stiffness which explains the initial increasing trend. For large enough values of $\frac{{E}_{h}}{{E}_{s}}$, however, the hard phase is so much stiffer than the soft phase that it practically behaves as a rigid material. Therefore, a further increase in ${E}_{h}$ does not noticeably affect the stress flow in the lattice structure and eventually, the degree of non-affinity saturates. In other words, up to a certain value of $\frac{{E}_{h}}{{E}_{s}}$, the deformation experienced by heterogeneous structures increasingly deviates from the one experienced by equivalent homogeneous structures.

The degree of non-affinity increased with ${\rho }_{h}$ until a maximum value was reached for ${\rho }_{h}=75\%-90\%$ (Fig. 3b-top). For the larger values of ${\rho }_{h}$, the degree of non-affinity is decreased such that it reaches $\Gamma$ = 0 for ${\rho }_{h}=100\mathrm{\%}$ (i.e.*,* a monolithically hard material), (Fig. 3b-top). The maximum value of $\Gamma$ occurred around the same ${\rho }_{h}$ value regardless of the type of the unit cell and the level of the applied strain (Fig. 3b-top). The same general trends were also valid for the higher levels of applied strain (e.g., 10%) (Fig. 3b-bottom). The plot of $\Gamma$ vs. ${\rho }_{h}$ was therefore highly asymmetric in all cases considered here (Fig. 3b). The initial increase in $\Gamma$ as ${\rho }_{h}$ increased is expected given that a higher ${\rho }_{h}$ value translates into a larger number of highly stiff struts that block the deformation of their surrounding low-stiffness struts. Moreover, the high stiffness struts can more effectively affect the stress flow in the lattice structures made from mostly low stiffness struts than the other way around. This explains the asymmetry in the plot of $\Gamma$ vs. ${\rho }_{h}$.

**Figure 4:**
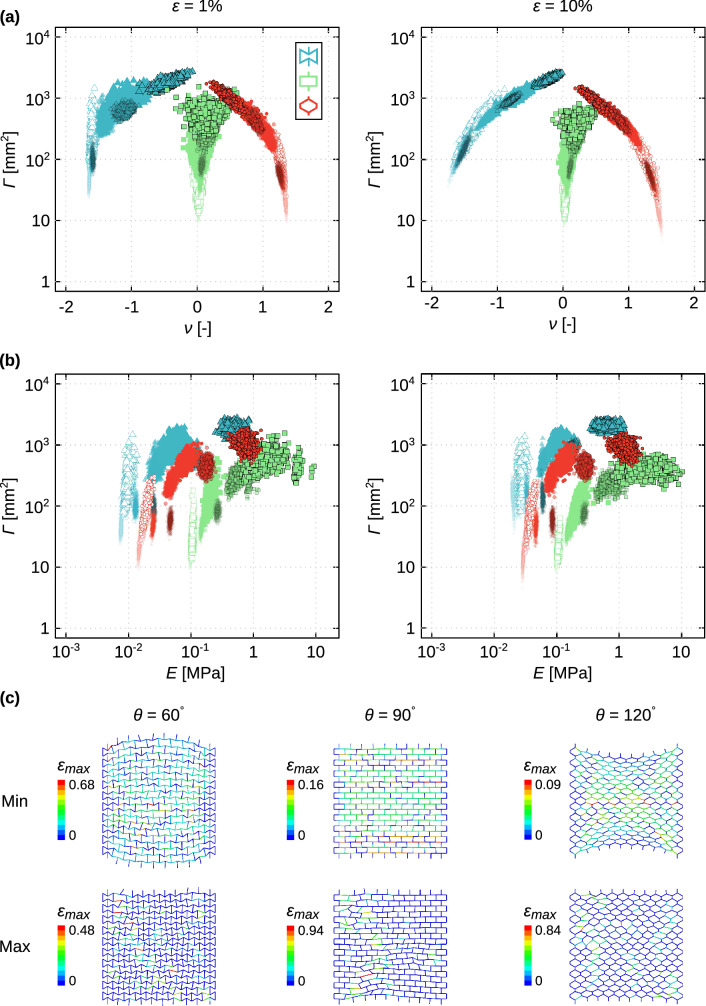
The changes in the degree of non-affinity as functions of the Poisson’s ratio (**a**) and the elastic modulus (**b**) at two levels of the applied strain (1% and 10%). A selected number of cases are depicted in (**c**), representing the lattice structures with the minimum and maximum values of $\Gamma$ with $\frac{{E}_{h}}{{E}_{s}}=1000$. The design parameters for each of the specimens presented in (**c**) are listed in Table S6 of the supplementary document. The color bars in subfigure (**c**) show the maximum strain distribution in %.

For both auxetic and honeycomb unit cells, there was a very clear (power-law) relationship between the Poisson’s ratio and the degree of non-affinity of the lattice structures (Figure S2 of the supplementary document). In general, the degree of non-affinity was up to 2 orders of magnitude lower for the lattice structures with the extreme absolute values of the Poisson’s ratio (Fig. 4a-left). This relationship was even stronger (i.e., less scatter around the power-law trend line) for the higher levels of applied strain (Fig. 4a-right, and Figure S2 of the supplementary document). No such relationship was, however, observed for the lattice structures with near-zero Poisson’s ratios (i.e., $\theta =90^\circ$) (Fig. 4a). These observations explain that achieving highly negative and highly positive values of the Poisson’s ratio requires that all or most of the struts contribute towards the targeted type of deformation. A homogenous (i.e.*,* highly affine) distribution of the deformations among the different unit cells of the lattice structure is particularly efficient in achieving large lateral deformations that are needed for large absolute values of the Poisson’s ratio. That is because similar deformations exhibited by all unit cells add up instead of (partially) canceling each other out (Fig. 4c).

The relationship between the elastic modulus and the degree of non-affinity was less clear (Fig. 4b). For each type of the unit cells, the degree of non-affinity was generally higher for the stiffer lattice structures (Fig. 4b). This, however, attributes to the fact that a higher value of ${\rho }_{h}$ both increases the degree of non-affinity and the stiffness of the lattice structure.

In the present study, we excluded the geometrical and topological complexities that are relevant for the design of mechanical metamaterials. Those parameters have shown to influence the degree of non-affinity^33^. In addition, we used a limited number of unit cells (i.e.*,* 10 × 10) to minimize the effects of boundary conditions on our computational models. We believe increasing the number of unit cells will not change the trend of non-affinity that we found here. However, the effects of geometrical and topological parameters on non-affinity need to be further studied.

## Conclusion

We studied here, for the first time in the area of extremal mechanical metamaterials, the non-affinity of the deformations experienced by multi-material mechanical metamaterials with random distributions of a hard phase within a lattice structure made of a soft material. We isolated the effects of multi-material design from those of geometry by comparing the deformation observed in our lattice structures with those of geometrically identical lattice structures that were made from one single material. Our results clearly show that a multi-material design approach could lead to both a wide range of elastic properties and a wide range of non-affine deformations. We found that the degree of non-affinity is strongly correlated with the design parameters including $\theta$, ${\rho }_{h}$, and $\frac{{E}_{h}}{{E}_{s}}$. In addition, the degree of non-affinity is highly correlated to the mechanical properties particularly the Poisson’s ratio. Interestingly, achieving extremely high levels of auxeticity (or highly positive Poisson’s ratios) seems to require highly affine deformations in multi-material mechanical metamaterials. On the other hand, achieving high values of the elastic modulus with multi-material mechanical metamaterials is associated with high levels of non-affine deformations. This is a new type of incompatibility between the very high values of the elastic modulus and very high absolute values of the Poisson’s ratio. It is important to realize that this incompatibility is different from the other types of such incompatibilities observed in the past (e.g., see^46^), as this incompatibility pertains to the spatial distribution of the mechanical properties within the lattice structure and not to the geometrical design of the unit cells (i.e., bending-dominated vs. stretch-dominated unit cells). The high levels of non-affinity observed here for multi-material mechanical metamaterials are expected to have clear implications for the structural integrity of the lattice structures. That is due to the high level of stress concentrations that are created as a result of such non-affine deformations. The high-stress concentration zones could accelerate crack initiation and propagation and ultimately lead to premature structural failure. The use of functional gradients may, therefore, be required to mitigate the structural effects of non-affine deformations.

## Supplementary information

Supplementary information
