# Supplementary material for: Non-affinity in multi-material mechanical metamaterials
Source: Sci Rep. 2020 Jul 13;10:11488. doi: 10.1038/s41598-020-67984-6 (PMC7359350; doi:10.1038/s41598-020-67984-6)
Supplement: Supplementary file 1 — Supplementary information [file 41598_2020_67984_MOESM1_ESM.docx]

Supplementary document to

Non-affinity in multi-material mechanical metamaterials

M. J. Mirzaali^a,^^[[1]](#footnote-1)^, H. Pahlavani^a^, E. Yarali^b^, A. A. Zadpoor^a^

*^a^ Department of Biomechanical Engineering, Faculty of Mechanical, Maritime, and Materials Engineering, Delft University of Technology (TU Delft), Mekelweg 2, 2628 CD, Delft, The Netherlands*

*^b^ School of Mechanical Engineering, College of Engineering, University of Tehran, Tehran, Iran*

**
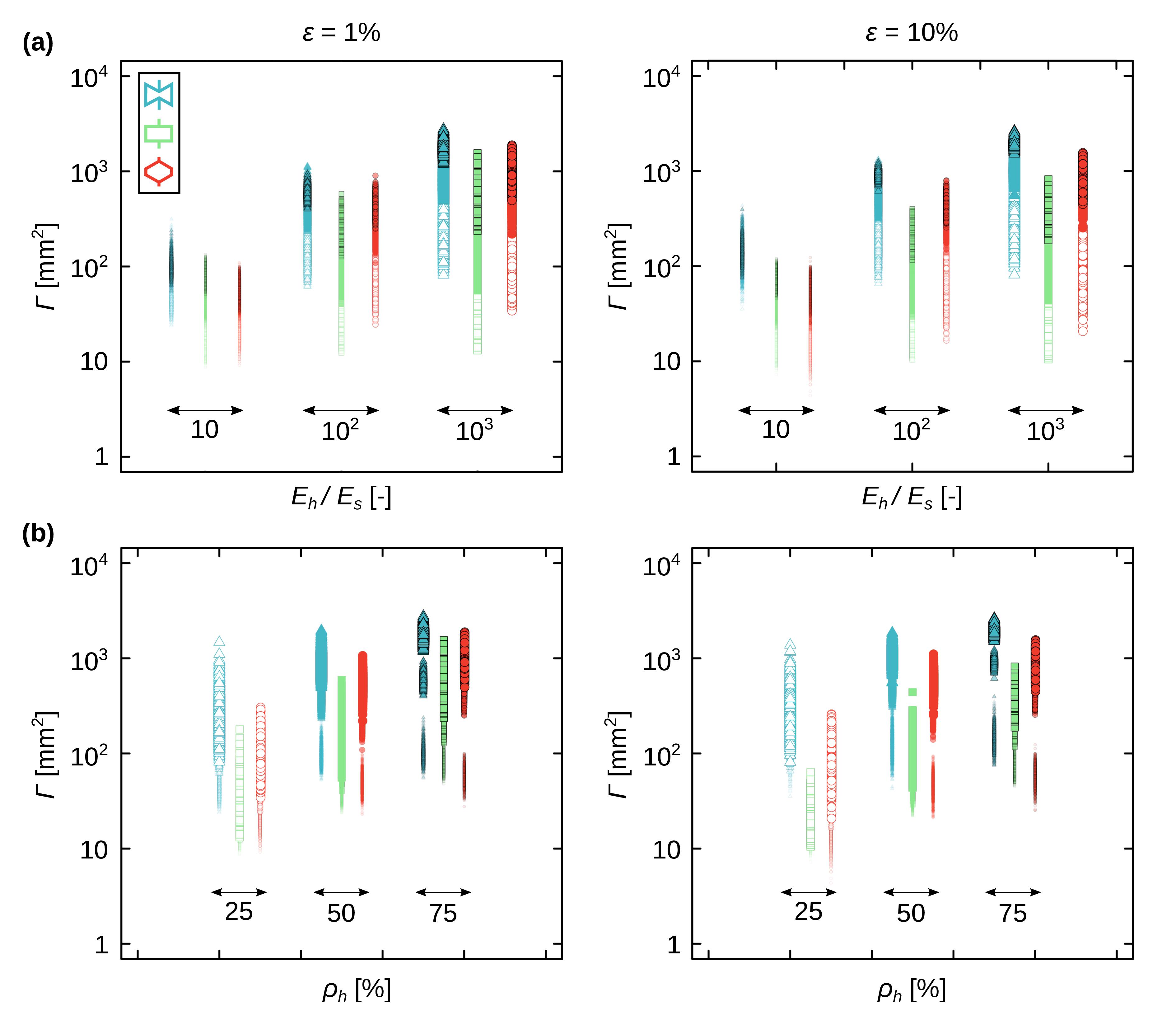
Figure S1.** The changes in the degree of non-affinity with $\frac{E_{h}}{E_{s}}$ (a) and the volume fraction of the hard material, $\rho_{h}$, (b) for the three different types of unit cell geometries and two levels of the applied strain (*i.e.*, 1% and 10%). Pooled data (*i.e.,* $\rho_{h}=$25%, 50% and 75%, and $\frac{E_{h}}{E_{s}}=$10, 100, and 1000) are shown in this figure.

**
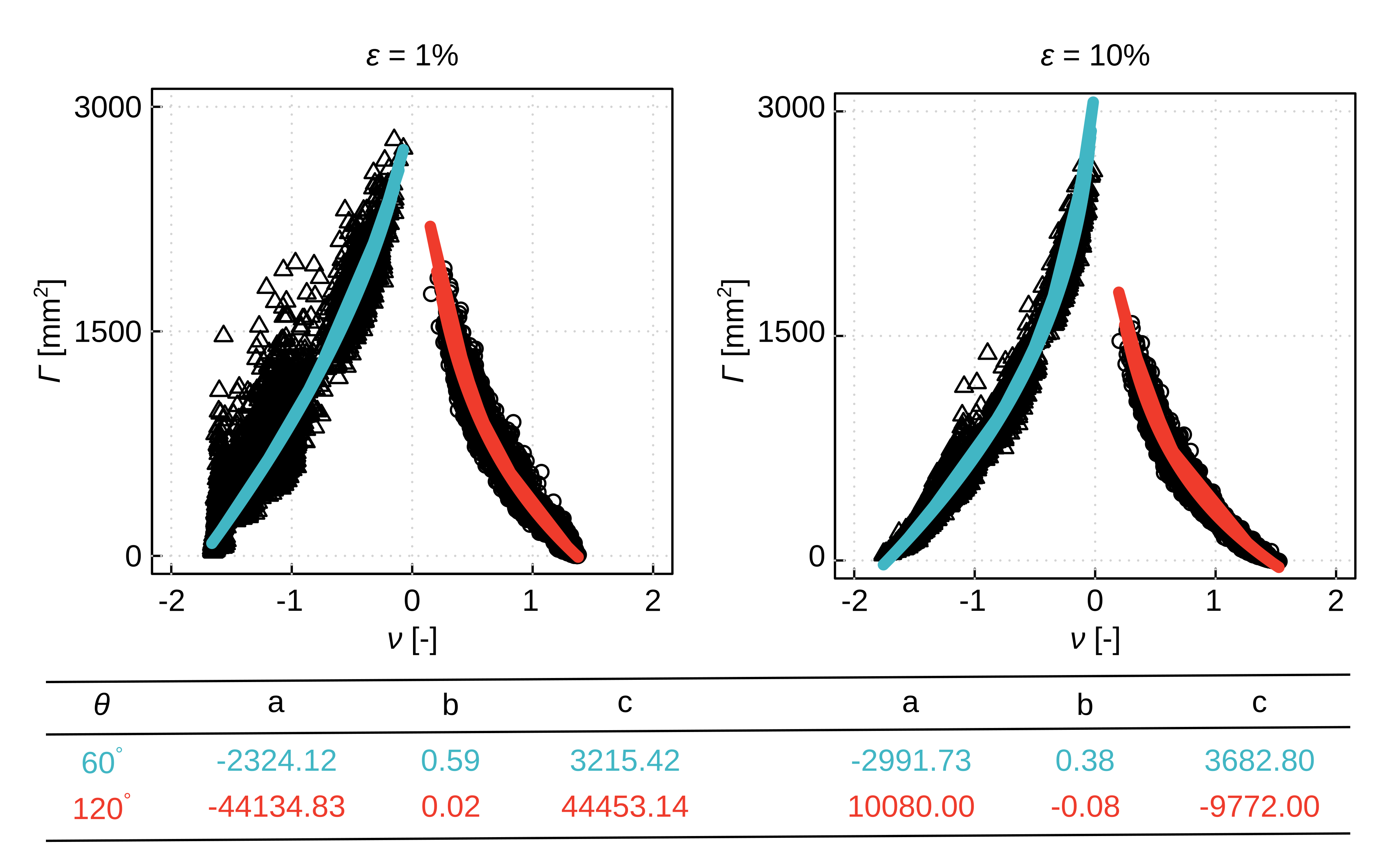
**

**Figure S2.** Both for the auxetic and honeycomb types of the unit cells, the degree of non-affinity and the Poisson’s ratio were related to each other through a power law (*i.e.*, $\Gamma=a\upsilon^{b}+c$*)*. The parameters of the fits for both types of the geometries are presented as well. The pooled data (*i.e.,* $\rho_{h}=$25%, 50% and 75%, and $\frac{E_{h}}{E_{s}}=$10, 100, and 1000) are presented in this figure.

**Figure S3.** A schematic of applied kinematic coupling constraints (a) and boundary conditions (b) in our computational models.

**Figure S4.** The distribution of the von-Mises stress in the selected designs presented in Figure 2c. The hard-soft stiffness ratio was set to 1000. The presented results correspond to an applied strain of 10%.

**Table S1.** The design parameters of the lattice structures.

| $\theta[^{\circ}]$ | $W[mm]$ | $L[mm]$ | $w[mm]$ | $h[mm]$ | $l[mm]$ | $c[mm]$ | $t[mm]$ | $b[mm]$ |
| --- | --- | --- | --- | --- | --- | --- | --- | --- |
| 60 | 112.5 | 124.3 | 11.25 | 9.17 | 6.50 | 11.85 | 0.75 | 7.5 |
| 90 | 112.5 | 124.3 | 11.25 | 5.92 | 5.63 | 11.85 | 0.75 | 7.5 |
| 120 | 112.5 | 124.3 | 11.25 | 2.67 | 6.50 | 11.85 | 0.75 | 7.5 |

**Table S2.** A comparison between the experimentally measured (Exp) and computationally determined (FEA) values of the elastic properties (*i.e*., elastic modulus and Poisson’s ratio) with $\frac{E_{h}}{E_{s}}=1000$. The data are reported at 1% strain.

| $\theta[^{\circ}]$ | $\rho_{h} [\%]$ | $E_{Exp} [MPa]$ | $E_{FEA} [MPa]$ | $\nu_{Exp} [-]$ | $\nu_{FEA}[-]$ | $\Gamma[{mm}^{2}]$ |
| --- | --- | --- | --- | --- | --- | --- |
| 60 | 25 | 0.03 ± 0.002 | 0.01 | -1.31 ± 0.08 | -1.61 | 400.41 |
| 90 | 75 | 0.77 ± 0.13 | 1.14 | 0.01± 0.06 | -0.06 | 402.36 |
| 120 | 50 | 0.10 ± 0.01 | 0.09 | 0.89 ± 0.05 | 0.99 | 395.00 |

**Table S3.** The parameters of the Gaussian distributions (*i.e*., mean and standard deviation (SD)) and the gamma distributions (*i.e.,* shape, rate, and skewness) of the non-affinity parameter, $\Gamma[{mm}^{2}]$ presented in Figure 2a and Figure S1a. The pooled data (*i.e.,* $\rho_{h}=$25%, 50% and 75%) are presented in this table.

|  |  | $\varepsilon[\%]$ =1 | | | | | $\varepsilon[\%]$ =10 | | | | |
| --- | --- | --- | --- | --- | --- | --- | --- | --- | --- | --- | --- |
| $\theta[^{\circ}]$ | $\frac{E_{h}}{E_{s}}$ | mean | SD | Shape | Rate | Skewness | Mean | SD | Shape | Rate | Skewness |
| 60 | 10 | 86.6 | 34.6 | 6.0 | 0.1 | 0.8 | 145.6 | 52.3 | 8.3 | 0.1 | 0.7 |
| 90 | 10 | 47.0 | 29.0 | 2.4 | 0.1 | 1.3 | 42.9 | 26.6 | 2.4 | 0.1 | 1.3 |
| 120 | 10 | 44.0 | 19.1 | 4.6 | 0.1 | 0.9 | 41.4 | 21.7 | 3.0 | 0.1 | 1.2 |
| 60 | 100 | 411.3 | 200.9 | 3.2 | 0.0 | 1.1 | 585.2 | 291.2 | 3.2 | 0.0 | 1.1 |
| 90 | 100 | 133.6 | 115.5 | 1.2 | 0.0 | 1.8 | 104.0 | 92.4 | 1.2 | 0.0 | 1.8 |
| 120 | 100 | 256.7 | 167.7 | 1.9 | 0.0 | 1.5 | 276.1 | 176.1 | 1.7 | 0.0 | 1.5 |
| 60 | 1000 | 1075.9 | 666.0 | 1.9 | 0.0 | 1.4 | 1218.7 | 693.7 | 2.2 | 0.0 | 1.4 |
| 90 | 1000 | 244.7 | 247.9 | 0.9 | 0.0 | 2.1 | 179.3 | 178.4 | 0.9 | 0.0 | 2.1 |
| 120 | 1000 | 586.8 | 443.0 | 1.3 | 0.0 | 1.7 | 534.2 | 376.6 | 1.3 | 0.0 | 1.7 |

**Table S4.** The parameters of the Gaussian distributions (*i.e*., mean and standard deviation (SD)) and the gamma distributions (*i.e.,* shape, rate, and skewness) of the non-affinity metric $\Gamma[{mm}^{2}]$ presented in Figure 2b and Figure S1b. The pooled data ($\frac{E_{h}}{E_{s}}=$10, 100, and 1000) are presented in this table.

|  |  | $\varepsilon[\%]$ =1 | | | | | $\varepsilon[\%]$ =10 | | | | |
| --- | --- | --- | --- | --- | --- | --- | --- | --- | --- | --- | --- |
| $\theta[^{\circ}]$ | $\rho_{h} [\%]$ | mean | SD | Shape | Rate | Skewness | mean | SD | Shape | Rate | Skewness |
| 60 | 25 | 162.9 | 135.8 | 1.8 | 0.0 | 1.5 | 247.1 | 148.0 | 3.4 | 0.0 | 1.1 |
| 90 | 25 | 24.0 | 10.8 | 6.7 | 0.3 | 0.8 | 18.8 | 5.8 | 11.7 | 0.6 | 0.6 |
| 120 | 25 | 65.3 | 43.7 | 2.2 | 0.0 | 1.3 | 58.3 | 41.6 | 1.8 | 0.0 | 1.5 |
| 60 | 50 | 485.9 | 363.8 | 1.5 | 0.0 | 1.6 | 590.5 | 406.6 | 1.6 | 0.0 | 1.6 |
| 90 | 50 | 95.3 | 58.6 | 3.2 | 0.0 | 1.1 | 69.5 | 35.7 | 4.6 | 0.1 | 0.9 |
| 120 | 50 | 286.0 | 225.3 | 1.3 | 0.0 | 1.7 | 306.5 | 230.1 | 1.3 | 0.0 | 1.8 |
| 60 | 75 | 853.0 | 745.8 | 1.0 | 0.0 | 2.0 | 1034.1 | 774.7 | 1.2 | 0.0 | 1.8 |
| 90 | 75 | 306.1 | 220.7 | 1.8 | 0.0 | 1.5 | 237.8 | 151.1 | 2.3 | 0.0 | 1.3 |
| 120 | 75 | 533.6 | 458.8 | 1.0 | 0.0 | 2.0 | 485.1 | 383.7 | 1.1 | 0.0 | 1.9 |

**Table S5.** The specifications of the sample finite element models depicted in Figure 2c. The hard-soft stiffness ratio (*i.e.,* $\frac{E_{h}}{E_{s}}$) was 1000.

| $\theta[^{\circ}]$ | $\rho_{h} [\%]$ | $\Gamma[{mm}^{2}]$ | $\nu[-]$ | $E[MPa]$ |
| --- | --- | --- | --- | --- |
| 60 | 25 | 345.57 | -1.31 | 0.03 |
|  | 50 | 1087.50 | -0.75 | 0.08 |
|  | 75 | 2014.10 | -0.31 | 0.47 |
| 90 | 25 | 22.30 | 0.03 | 0.11 |
|  | 50 | 102.49 | 0.06 | 0.20 |
|  | 75 | 412.77 | 0.28 | 2.57 |
| 120 | 25 | 94.60 | 1.24 | 0.04 |
|  | 50 | 575.24 | 0.75 | 0.12 |
|  | 75 | 954.31 | 0.51 | 1.10 |

**Table S6.** The specifications of the sample finite element models depicted in Figure 4c. The hard-soft stiffness ratio (*i.e.,* $\frac{E_{h}}{E_{s}}$) was 1000.

| $\theta[^{\circ}]$ | $\rho_{h} [\%]$ | $\Gamma[{mm}^{2}]$ | $\nu[-]$ | $E[MPa]$ |
| --- | --- | --- | --- | --- |
| 60 | 25 | 81.24 | -1.65 | 0.03 |
|  | 75 | 2639.14 | -0.11 | 0.67 |
| 90 | 25 | 10.52 | 0.04 | 0.10 |
|  | 75 | 816.69 | 0.25 | 1.83 |
| 120 | 25 | 21.24 | 1.44 | 0.04 |
|  | 75 | 1596.61 | 0.30 | 1.11 |

1. Corresponding author. Tel.: +31-15-2783133*, e-mail:* [m.j.mirzaalimazandarani@tudelft.nl](mailto:m.j.mirzaalimazandarani@tudelft.nl) [↑](#footnote-ref-1)
